# Bark investment is key to forest expansion into African savannas by conferring resistance to fire and seasonal drought

**DOI:** 10.1093/aob/mcaf019

**Published:** 2025-05-02

**Authors:** Julieta A Rosell, Susanne Vetter, Mark E Olson, Michelle Greve

**Affiliations:** Laboratorio Nacional de Ciencias de la Sostenibilidad, Instituto de Ecología, Universidad Nacional Autónoma de México, A.P. 70-275, Ciudad Universitaria, Coyoacán, Mexico City 04510, Mexico; Department of Botany, Rhodes University, Makhanda 6140, South Africa; Department of Botany, Rhodes University, Makhanda 6140, South Africa; Department of Botany, Rhodes University, Makhanda 6140, South Africa; Instituto de Biología, Universidad Nacional Autónoma de México, A.P. 70-275, Ciudad Universitaria, Coyoacán, Mexico City 04510, Mexico; Department of Plant and Soil Sciences, University of Pretoria, Pretoria 0028, South Africa

**Keywords:** Bark, drought resistance, fire resistance, forest expansion, inner bark, outer bark, savanna encroachment, secondary phloem, water storage, wood density, woody encroachment

## Abstract

**Background and Aims:**

Forest expansion into savannas is widespread even though fire and seasonal drought provide environmental conditions against encroachment by forest specialists. A distinct suite of species can establish under savanna trees, forming bush clumps and facilitating forest establishment. Understanding the functional traits of clump-forming species is crucial for uncovering encroachment mechanisms and devising management strategies. Bark likely plays a key role in enabling clump initiation. Fire resistance can be achieved by accumulation of outer bark thickness (OBT), height and/or stem diameter (SD), while drought resistance may be enhanced by greater inner bark thickness (IBT), associated with water and carbohydrate storage.

**Methods:**

We selected representative savanna, clump-forming and closed-canopy species (ecological categories) at two South African sites experiencing forest expansion and differing in rainfall and fire frequency. We compared OBT–SD and IBT–SD allometries across ecological categories and sites and examined whether categories separated along axes reflecting fire/drought resistance (OBT and IBT) and resource allocation strategy (density and water content, leaf size).

**Key Results:**

OBT–SD scaling of clump-forming species was more similar to savanna than forest species, and savanna species at the more fire-prone savanna had steeper OBT–SD scaling, consistent with high OBT providing fire protection in early clump formation. Similar IBT–SD slope across groups was consistent with similar metabolic needs, while higher intercepts in savanna and clump-forming species indicated higher water storage. ‘Cheap’ low-density tissues in savanna species allow fast accumulation of SD and OBT and resistance to fire topkill. Closed-canopy species had denser tissues and thin stems and bark for a given height, while the clump-forming species were intermediate.

**Conclusions:**

Bark and probably other traits are key in the capacity of some species to form bush clumps. Identifying these traits and the mechanisms underlying clump formation is essential for managing encroached savannas and grasslands.

## INTRODUCTION

Woody encroachment in grasslands, savannas and other open vegetation has been documented globally (Stevens *et al.*, 2017). Encroachment (when shrubs and trees replace or reduce the dominance of grasses and herbaceous vegetation in savannas or grasslands) is promoted by diverse factors, including increased rainfall, decreased rainfall seasonality, fire suppression, loss of large browsers, and CO_2_ fertilization (Stevens *et al.*, 2016; Skowno *et al.*, 2017; Venter *et al.*, 2018). Woody encroachment can take the form of savanna densification or forest expansion (Parr *et al.*, 2014), the latter involving a compositional and functional shift from open vegetation with sparse trees and shrubs and a continuous grassy layer that is prone to frequent fires, to a system with abundant trees and shrubs forming a closed canopy that excludes fire-promoting grasses (Cardoso *et al.*, 2018; Charles-Dominique *et al.*, 2018). Forest expansion into savannas and grasslands is a pressing issue because it has important consequences for forage production, biodiversity and wildlife conservation, groundwater recharge and pastoral livelihoods (Parr *et al.*, 2014; Luvuno *et al.*, 2018; Briske and Coppock, 2023). A better understanding of the ecological processes involved in woody encroachment is essential in better predicting and managing these biome shifts that can occur in just 15 years (Jeffery *et al.*, 2014), and that are difficult and costly to reverse (Wilcox *et al.*, 2018).

Fire and seasonal drought create conditions that prevent the establishment of forest specialists within the savanna (Hoffmann *et al.*, 2004, 2012; Cardoso *et al.*, 2016). Forest expansion into savannas thus requires species that can establish within a savanna and create conditions more favourable for the establishment of less drought- and fire-tolerant forest species (Abreu *et al.*, 2021; Jamison-Daniels *et al.*, 2021). The transition of savanna to closed vegetation has been documented to occur via the formation of bush clumps in a variety of savanna systems. Bush clumps are clusters of shrubs or small trees that form localized patches of woody vegetation within a savanna or grassland ecosystem. During encroachment, isolated fire-tolerant trees facilitate the establishment of woody species that form dense clumps of woody vegetation comprising individuals of multiple species (Archer, 1995; O’Connor and Chamane, 2012; Abreu *et al.*, 2021; Jamison-Daniels *et al.*, 2021; Adie *et al.*, 2023; Nell *et al.*, 2024). As these bush clumps expand and eventually coalesce, the dense vegetation shades out the grass layer, inhibiting fire and creating a cooler, moister microclimate that facilitates the establishment of closed-canopy species that do not establish in the open (Hoffmann *et al.*, 2012; Cardoso *et al.*, 2018; Charles-Dominique *et al.*, 2018; Jamison-Daniels *et al.*, 2021). Encroachment appears to depend on a distinct suite of species able to initiate the formation of bush clumps and to survive in them (Barnes and Archer, 1996; O’Connor and Chamane, 2012; Nell *et al.*, 2024). This clump-forming capacity seems necessarily mediated by plant functional traits that confer tolerance of the fire and seasonal drought regimes that characterize savannas, albeit at a lower intensity due to the altered microclimate, fuel biomass and moisture usually found under the canopies of large savanna trees that typically serve as nuclei for clumps (Cardoso *et al.*, 2018; Abreu *et al.*, 2021). Identifying the traits conferring the capacity for bush clump formation is thus necessary to understand and manage woody encroachment.

Despite the importance of a functional trait perspective for understanding the mechanisms underlying clump formation, there have been no studies examining key traits of clump-forming species. Previous studies that focused on the characteristics of forest precursor species that colonize savannas or grow at savanna–forest ecotones have highlighted the importance of fire-related traits such as bark thickness (Hoffmann *et al.*, 2012; Flake *et al.*, 2021) and bud protection (Charles-Dominique *et al.*, 2015; Chiminazzo *et al.*, 2021), as well as starch storage and fast growth (Cardoso *et al.*, 2016). In addition to fire, drought has been highlighted as a significant factor limiting the establishment of trees in savannas (Cardoso *et al.*, 2016), as evidenced by forest-associated species establishing late in the clump formation process once the microclimate has become ameliorated by trees (Jamison-Daniels *et al.*, 2021). Testing whether bark and other functional traits of clump-forming species are more similar to savanna than to species of forests or other closed-canopy vegetations would identify crucial traits underlying the initiation phase of thicket or forest expansion.

Bark is likely to represent a crucial stem region for species establishing during the initiation phase of a clump. Total bark includes all the tissues outside the vascular cambium, and it can be divided from a functional perspective into two regions, the outer bark (OB), which includes dead protective tissues, and the inner bark (IB), which includes mostly living tissues (Romero, 2014; Rosell, 2019). While most previous studies have focused on total bark thickness as the trait protecting stems from the necrosis and hydraulic failure caused by fire (Michaletz, 2018), increasing evidence suggests that it is OB that is shaped by natural selection in protecting against fire in fire-prone habitats (Graves *et al.*, 2014; Schafer *et al.*, 2015; Rosell, 2016; Scalon *et al.*, 2021). In turn, IB carries out photosynthate transport, as well as water, carbohydrate and nutrient storage, and in many cases photosynthesis (Loram-Lourenço *et al.*, 2020; Rosell *et al.*, 2021, 2023; Scalon *et al.*, 2021) (Fig. 2). Accordingly, IB tends to be thicker in species of more seasonal and hotter sites, likely buffering changes in water availability (Scalon *et al.*, 2021). Thus, both OB and IB could make important contributions to clump-forming ability, with thick OB providing protection against fire and thick IB increasing tolerance to seasonal drought and fuelling resprouting after disturbance.

**Fig. 1.**
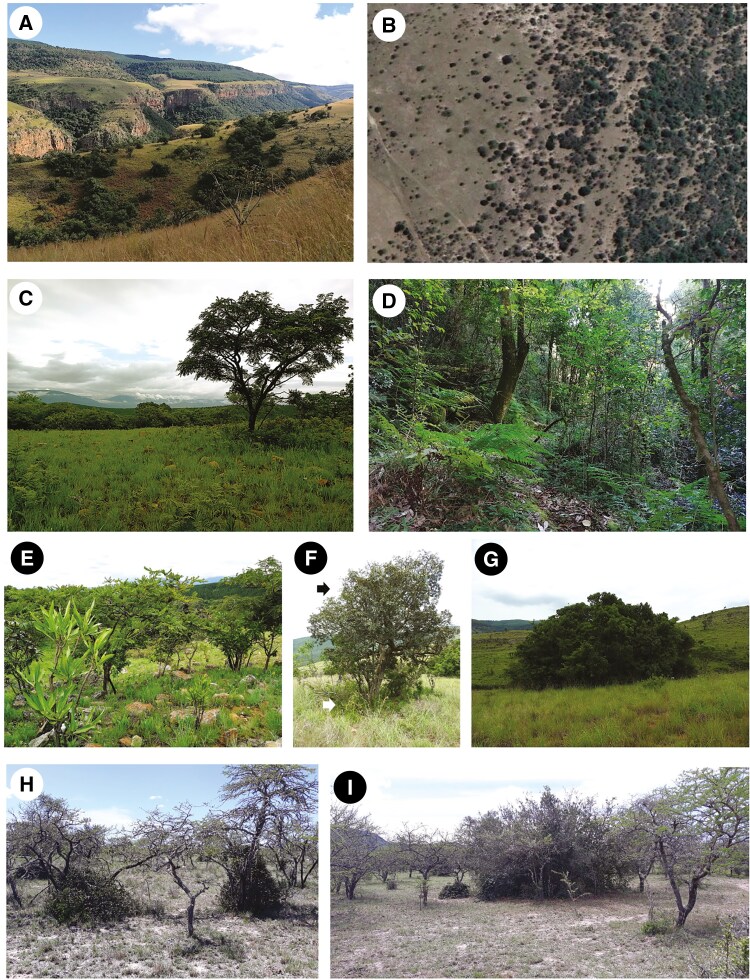
Bush clump formation at the two study sites. (A) Bush clump formation in savanna near Sudwala with forest in the background. (B) Bush clump formation at Endwell showing open savanna with solitary savanna trees to the left and increasing size and density of bush clumps to the right. Credit: Google Earth. (C) Savanna at Sudwala. (D) Forest, Sudwala. (E) Savanna saplings in Sudwala showing establishment within recently burned grassy matrix. (F) Clump-forming species (white arrow) establishing under a savanna tree (black arrow) in Sudwala. (G) Established and expanding bush clump at Sudwala. (H) Early stages of bush clump formation at Endwell showing recruitment of *Scutia myrtina* under *Vachellia karroo*. (I) Bush clumps of different sizes interspersed with solitary savanna trees (*V. karroo*) at Endwell.

Bark thickness is strongly predicted by stem diameter (SD), but there is significant variation in the slope and intercept of the bark thickness–SD scaling relationship (log–log scale; Rosell *et al.*, 2017). An approach taking this scaling into account is crucial to understand the role of OB or IB in the clump-forming capacity of a species (Rosell, 2016). For example, in resisting frequent surface fires, clump-forming species would be expected to have a higher rate of bark accumulation (Charles‐Dominique *et al.*, 2017) and hence thicker OB for a given SD than closed-canopy species that lack this encroaching capacity. Also, greater relative OB investment, and thus steeper OB thickness–SD slopes, would be expected in clump-forming species encroaching mesic savannas with more frequent fires fuelled by higher precipitation and grass productivity in comparison with semi-arid savannas (Govender *et al.*, 2006).

In addition to fire, seasonal drought has a strong effect on savanna species (Hoffmann *et al.*, 2004), and species in clumps must also face drier conditions in comparison with closed-canopy environments (Jamison-Daniels *et al.*, 2021). These drier conditions cause the greatest attrition per growing season at seedling and sapling phases (Cardoso *et al.*, 2016), and are thus expected to present an important selective pressure on individuals initiating a bush clump, especially during the dry season. Inner bark likely has an important, but unexamined role in the establishment of clump-forming species. Inner bark is made up mainly of the secondary phloem, the cortex and the phelloderm, all of which have a large fraction of parenchyma, a tissue specialized in storage (Plavcová and Jansen, 2015). Congruently, IB can store large amounts of water and non-structural carbohydrates (Loram-Lourenço *et al.*, 2020; Rosell *et al.*, 2021), crucial compounds in the process of reversing embolism (Nardini *et al.*, 2011), a phenomenon likely common in small plants during early clump formation. Establishment in drier environments is thus likely facilitated by higher allocation to IB for a given SD in clump-forming species in comparison with closed-canopy species. Given that IB is mainly made up of the secondary phloem, whose amount is expected to reflect metabolic needs, the IB–SD scaling relationship would be expected to have a similar slope across ecological categories, reflecting similar metabolic needs for a given SD (Rosell *et al.*, 2017), and to reflect higher storage needs through higher intercepts for savanna and clump-initiating species. By the same token, higher intercepts would also be predicted for clump-forming and savanna species of more arid savannas in comparison with less arid sites.

Along with higher allocation to OB, faster growth can aid in escaping the repeated topkill by fire that leads to a demographic bottleneck, with individuals persisting as short-statured plants, unable to reach canopy height and reproductive maturity (Freeman *et al.*, 2017). It has been suggested that slower growing savanna species should invest in thicker fire-protective OB (Scalon *et al.*, 2020), but with short fire intervals both strategies, faster growth and thicker OB, seem crucial and can be achieved for the same amount of carbon investment through lower density tissues (Olson *et al.*, 2018). Accordingly, frequent fire has been associated with faster growth rate, and thicker and moister bark in an African savanna (Higgins *et al.*, 2012). Like savanna species, clump-forming species must possess traits that avoid topkill, and they face the additional selection pressure of lower light availability in their subcanopy habitat. Their growth rates would be expected to be intermediate between savanna and forest species. Growth rates are embedded in a tight network of trait covariation (Chave *et al.*, 2009; Olson *et al.*, 2018). As a result, faster growing species should exhibit lower bark and wood density, higher bark and wood water contents, and also larger leaf sizes (Rosell *et al.*, 2014; Olson *et al.*, 2018; Loram-Lourenço *et al.*, 2020). Selection on any one of these traits would be expected to cause correlated changes in the others. As a result, understanding differences in bark traits between species almost certainly requires documenting covariation patterns among major functional traits.

To examine the role of bark and related traits in conferring clump-forming ability, we compared savanna, clump-forming and closed-canopy species (‘ecological categories’) at two sites in South Africa where thicket clump formation and expansion are occurring. The two selected savannas differ markedly in annual rainfall and fire frequency, allowing us to cover a wide range of phylogenetic and functional diversity across species and to test our hypotheses in two encroachment processes with differing fire and drought regimes. Examining the allocation patterns to IB and OB and their covariation with other bark, wood and leaf traits across the three ecological categories, we tested the following hypotheses: (1) OB allocation for a given SD is highest in savanna species and higher in clump-forming species than in closed-canopy species, suggesting that OB has an important role in fire protection during the encroachment process; (2) allocation to OB for a given SD is higher in clump-forming (and savanna) species from the mesic than arid savanna, reflecting more frequent and intense fire fuelled by higher precipitation and grass productivity, while OB allocation in (fire-free) closed-canopy vegetation does not differ between sites; (3) IB is thickest for a given SD in savanna species, and thicker in clump-forming species than closed-canopy species; (4) IB is thicker at the drier site when comparing within each ecological category, suggesting higher storage capacity of selective importance under lower precipitation; and (5) lower tissue density, higher water content and larger leaves are part of a trait syndrome in clump-forming and savanna species allowing thicker stems and thicker bark for the same amount of carbon, in comparison with forest species favoured by selection in achieving fast upward growth in shaded environments.

## MATERIALS AND METHODS

### Site and species selection

We selected two sites where savanna encroachment by bush clumps has been documented and the successional sequence has been characterized (Fig. 1). The two sites are markedly different in moisture availability and fire frequency. Moreover, they allowed us to examine 18 species representing a very wide range of phylogenetic lineages including 16 families in 13 taxonomic orders.

The first site is Endwell in the Eastern Cape, South Africa (32°45′5.08″S, 26°28′22.19″E; mean annual precipitation 730 mm), which includes a mosaic of semi-arid grassland, savanna and subtropical ‘thicket’ (often spiny shrub and small tree vegetation) on shale-derived, relatively eutrophic soils (Mucina and Rutherford, 2006). Woody expansion via bush clump formation has been documented at this site, with *Vachellia karroo* as the dominant savanna species that also serves as centre of aggregation for thicket clump formation by a distinct set of species (O’Connor and Chamane, 2012; Nell *et al.*, 2024).

The second site is Sudwala, Mpumalanga, South Africa (25°24′59.29″S, 30°40′37.57″E; mean annual precipitation 930 mm), which includes a mosaic of mesic savanna and forest on dystrophic sandstone-derived soils (Mucina and Rutherford, 2006). Sudwala was selected because it is near Buffelskloof Nature Reserve (BKNR), an area for which bush clump formation leading to forest expansion has been very well documented and in which the kind of sampling we needed to carry out was not possible. Sudwala and BKNR share species composition. At BKNR, and we assume at adjacent Sudwala, bush clump formation occurs under different species of savanna trees (and occasionally species that primarily occur in bush clumps rather than savannas, but never forest species) acting as founder individuals that facilitate the establishment of bush clumps.

In comparison with Endwell, Sudwala has markedly higher annual precipitation, but also a drier dry season (Supplementary Data Fig. S1). Historically, Sudwala would have had more frequent and intense fires than Endwell because of its higher rainfall and greater grass biomass (Govender *et al.*, 2006; Archibald and Hempson, 2016). In recent decades the savanna vegetation in Endwell has had a fire return interval of >10 years as farmers are reluctant to waste grass forage, while in Sudwala the savanna vegetation surrounding the bush clumps is subjected to biannual planned fires. The continuous forest or thicket vegetation at both sites is fire-free.

We selected eight species at Endwell and ten at Sudwala that are representative of one of three ecological categories: savanna, clump-forming and obligate closed-canopy (thicket or forest) species (Table 1). The selection of species was based on O’Connor and Chamane (2012) and Nell *et al.* (2024) for Endwell, on Jamison-Daniels *et al.* (2021) for Sudwala, and on field observations at each site. The ecological category was based on the habitat where each species was represented by the full range of size classes, including the smallest ones (indicating recruitment). Savanna species were those that were found to recruit and occur as single individuals in the grassy matrix. Closed-canopy species were those that recruited and matured under closed canopy woody vegetation (subtropical thicket at Endwell, forest at Sudwala) or inside very large bush clumps. Clump-forming species were most commonly associated as new recruits with mature savanna trees and at maturity occurred mainly in small thicket clumps or on the edges of large clumps. These clump-forming species can be considered generalists in the sense that they occur in different habitats, including savannas, the margins of closed-canopy vegetation (thicket or forest), and rocky areas. Here, we use the category ‘clump-forming species’ instead of ‘generalists’ to emphasize their role in the dynamic of clump formation. The number of savanna, clump-forming and closed-canopy species in our sample reflects the abundance of species from each category at each site, as we included only those species where the full range of size classes occurred in the preferred habitat.

**Table 1.** Site, family and ecological category for each sampled species

Site	Ecological category	Species	Family
Endwell	Savanna	*Vachellia karroo*	Fabaceae
	Clump-forming	*Brachylaena elliptica*	Asteraceae
		*Gymnosporia buxifolia*	Celastraceae
		*Olea europaea* subsp. *africana*	Oleaceae
		*Scutia myrtina*	Rhamnaceae
		*Ziziphus mucronata*	Rhamnaceae
	Thicket (closed-canopy)	*Afrocanthium mundianum*	Rubiaceae
		*Zanthoxylum capense*	Rutaceae
Sudwala	Savanna	*Annona senegalensis*	Annonaceae
		*Cussonia spicata*	Araliaceae
		*Dombeya rotundifolia*	Malvaceae
		*Faurea rochetiana*	Proteaceae
		*Lannea discolor*	Anacardiaceae
	Clump-forming	*Euclea crispa*	Ebenaceae
		*Searsia pentheri*	Anacardiaceae
		*Syzygium cordatum*	Myrtaceae
	Forest (closed-canopy)	*Celtis africana*	Cannabaceae
		*Combretum kraussii*	Combretaceae

### Bark and xylem traits

We selected 24–29 individuals per species, ranging from very small saplings (1 mm in basal diameter) to the largest adults for SD–bark thickness allometries. All bark thickness and SD measurements were made close to the base of the plant, above any basal swelling, and avoiding stems with visible bark damage. We ensured a particularly good coverage of small sizes given that variables were log-transformed. For medium-sized or large individuals, we collected a 10 × 3 cm wedge of bark and wood at the base using a saw and a screwdriver. For small individuals, we cut whole stem segments 5–10 cm in length at the base. At the sampling point, we measured SD using digital callipers or a tape measure. We also measured the height of each individual as the length from the base to the furthest branch tip.

We measured total bark (TB) and IB thickness on samples using digital callipers or on thin sections using a microscope when TB and IB were very thin (e.g. in young stems). We calculated OB thickness as the difference between TB and IB. We identified IB based on colour, texture, moisture and cell types, using thin sections under a microscope when needed (Angyalossy *et al.*, 2016). Some saplings had not developed secondary growth. In these cases we considered all tissues outside the primary xylem as bark. In individuals in which secondary growth was present but the phellogen had not been activated, i.e. OB had not been yet produced, we considered OB to be functionally represented by the epidermis plus any external waxy layer. Only 2 % of our dataset was represented by saplings without the production of OB.

We measured the density and water content of IB, TB and sapwood for the three largest individuals of each species using 2 × 2 × 2 cm blocks. Blocks of IB were of variable depth depending on IB thickness. We saturated samples in water for 24 h before measuring fresh weight and volume. We measured volume using a balance and the water displacement method. We oven-dried samples at 80 °C until constant weight was reached. We calculated density as dried weight/fresh volume, and water content as (fresh weight − dried weight)/dried weight × 100 (Rosell *et al.*, 2014).

We quantified mean leaf size per species from voucher specimens collected at the sites, as well as from leaves from specimens at the Schonland and Buffelskloof Private Nature Reserve herbaria, totalling at least 30 leaves per species.

### Data analyses

We examined bark thickness–SD allometries using mixed linear models. For each site, we started by fitting a saturated model predicting outer bark thickness (OBT) based on SD (continuous variable) and ecological category (categorical variable with three levels: closed-canopy, clump-forming, savanna), and the interaction term between these two predictors. This interaction term allowed us to test for differences in the OBT–SD scaling across ecological categories. If this interaction term was not statistically significant, we removed it and refitted the model. For the random component, we nested observations from individual trees in species and allowed species to have random intercepts and slopes. The random component in all models was always significant and improved model fit. We log_10_ transformed continuous variables in all models and checked assumptions visually. We fitted a similar model for the pooled data of the two sites. In this case, we nested trees in species and species in sites in the random component. We applied the same fitting procedure to inner bark thickness (IBT) as the predicted variable. We fitted mixed models with the R package nlme (Pinheiro *et al.*, 2021).

To examine differences in bark thickness–SD allometries between sites, we also used mixed linear models. For each ecological category, we predicted OBT based on SD and site (Endwell or Sudwala), and their interaction. We followed the same procedure described above to fit the model. We fitted a similar model for IBT as the predicted variable.

To examine the network of trait covariation, we calculated bivariate correlations and carried out multivariate analyses. To compare bark allocation across species, we calculated IBT and OBT at the stem base for individuals 2 m in height per species. We chose this height to reflect the investment in bark by trees within flame height (Higgins *et al.*, 2012; Wigley *et al.*, 2020). To calculate IBT and OBT at 2 m, we first fitted a three-parameter exponential model (Hulshof *et al.*, 2015), which had the following equation: *H = a* − *b* × exp(*c × SD*), where *H* is height, *SD* is stem diameter, *a* is maximum asymptotic height, *b* is the difference between the maximum and minimum heights, and *c* is a curve-fitting parameter. We fitted these models using the R function nls and used them to calculate the SD associated with a height of 2 m. To calculate IBT and OBT for the estimated SD at 2 m tall, we fitted IBT–SD and OBT–SD models (variables log_10_ transformed) per species using simple linear regressions. Finally, we examined bivariate correlations on untransformed variables (except for leaf length, which was log_10_ transformed). Using estimated IBT and OBT at 2 m height (hereafter IBT_2m_ and OBT_2m_), we performed a principal component analysis that included wood and IB density and water content, and leaf length, using princomp in R. We performed all analyses in R v.4.3.1 (R Development Core Team, 2023).

## RESULTS

**Fig. 2.**
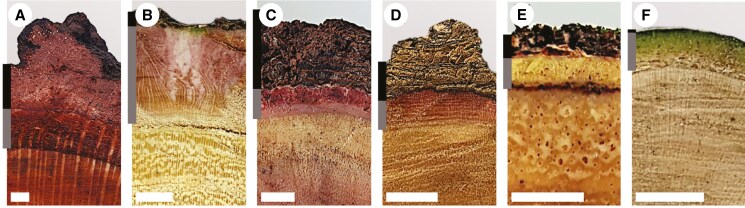
Bark and xylem from (A) *Faurea rochetiana* (Sudwala savanna), (B) *Vachellia karroo* (Endwell savanna), (C) *Searsia pentheri* (Sudwala clump-forming), (D) *Scutia myrtina* (Endwell clump-forming), (E) *Combretum kraussii* (Sudwala closed-canopy) and (F) *Afrocanthium mundianum* (Endwell closed-canopy). Grey bars indicate inner bark and black bars indicate outer bark. Scale bars: 5 mm.

The species sampled covered a wide range of variation in bark thickness (Fig. 2). Adults of closed-canopy species had very thin bark, representing <6 % of the trunk radius, mostly occupied by IB (70–90 % of total bark thickness, Fig. 2E, F). In contrast, adults of savanna species had markedly thicker bark, occupying up to 20 % of the trunk radius with OB representing 23–66 % of total bark (Fig. 2A, B). Most clump-forming species included adults with thick total bark (8–21 % of trunk radius) with varying proportions of IB and OB (Fig. 2C, D).

### Variation in outer bark allocation across ecological categories

**Fig. 3.**
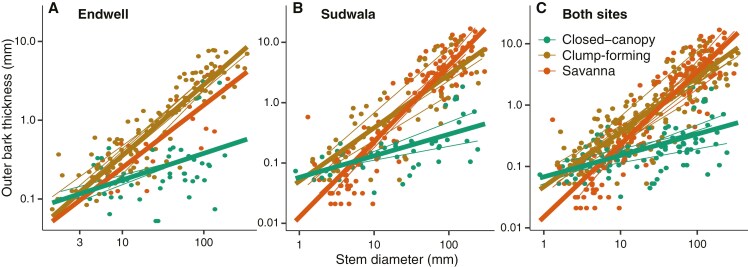
Mixed models predicting OBT based on SD and ecological category (closed-canopy, clump-forming, savanna) for (A) Endwell, (B) Sudwala and (C) both sites pooled. Thick lines represent general fits per ecological category and thin lines represent fits per species (considered as random factors). Parameters of models are included in Supplementary Data Table S1.

In mixed models, SD and ecological category were strong predictors of OBT (marginal and conditional *r*^2^ ≥ 0.74; Supplementary Data Table S1). OBT–SD slopes differed across ecological categories for Endwell, Sudwala and the two sites pooled, in the expected direction, supporting a strong role of OB in the capacity to form clumps and to persist under frequent fire (Fig. 3). Savanna species, which experience higher fire intensity, had higher slopes, but lower intercepts, than clump-forming species in Sudwala, although for Endwell slopes and intercepts did not significantly differ between savanna and clump-forming species. Fire-free closed-canopy species always had slopes that were lower than the other two categories (Supplementary Data Table S1).

**Fig. 4.**
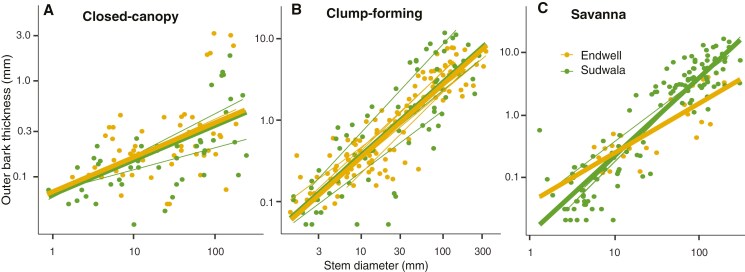
Mixed models predicting OBT based on SD and site (Endwell and Sudwala) for the different ecological categories: (A) closed-canopy, (B) clump-forming and (C) savanna. Thick lines represent general fits per site and thin lines represent fits per species (considered as random factors). Parameters of models are included in Supplementary Data Table S2.

Within each ecological category, we did not observe differences in OBT–SD scaling between sites, except for the species in the savanna set (Fig. 4, Supplementary Data Table S2). As predicted, the species of Sudwala, the site experiencing more frequent and intense fire, had higher OBT–SD slopes than Endwell, the semi-arid site. We had predicted that clump-forming species would also reflect site differences, but found no support for this (Fig. 4, Supplementary Data Table S2).

### Variation in inner bark allocation across ecological categories

**Fig. 5.**
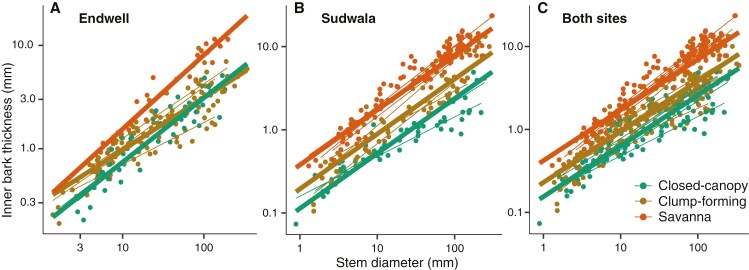
Mixed models predicting IBT based on SD and ecological category (closed-canopy, clump-forming, savanna) for (A) Endwell, (B) Buffelskloof and (C) both sites pooled. Thick lines represent general fits per ecological category and thin lines represent fits per species (considered as random factors). Parameters of models are included in Supplementary Data Table S3.

Mixed models for IBT–SD scaling had very good fits (marginal and conditional *r*^2^ ≥ 0.83; Supplementary Data Table S3). Our results were partially congruent with our expectation that IBT–SD scaling would reflect higher storage needs in savanna species, followed by clump-forming and closed-canopy species. At Sudwala, ecological categories did not differ in slope, but in intercept, in agreement with expectations, with savanna having the thickest IB for a given SD, and closed-canopy species the thinnest IB (Supplementary Data Table S3, Fig. 5B). IB–SD proportionality (slope) remained constant across plant sizes (Fig. 5B). For Endwell, categories differed in slope driven by the clump-forming group, which had the lowest slope strongly influenced by *Olea europaea* subsp. *africana* (Supplementary Data Table S3). When excluding this species, results were equivalent to Sudwala (Supplementary Data Table S4). When the species of the two sites were pooled, the results were again equivalent to those of Sudwala.

**Fig. 6.**
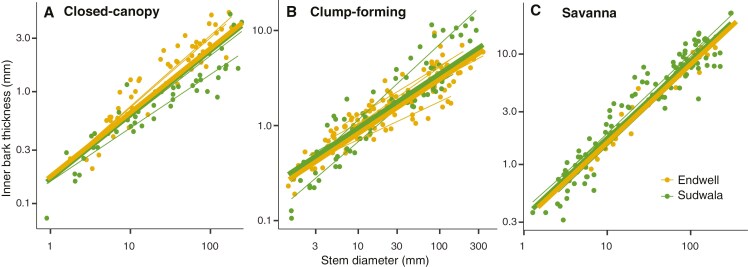
Mixed models predicting IBT based on SD and site (Endwell and Sudwala) for the different ecological categories: (A) closed-canopy, (B) clump-forming and (C) savanna species. Thick lines represent general fits per site and thin lines represent fits per species (considered as random factors). Parameters of models are included in Supplementary Data Table S5.

Within ecological categories, we did not find differences in IBT–SD scaling between sites (Supplementary Data Table S5, Fig. 6) for any ecological category.

### Covariation of allocation to inner and outer bark with other traits

The non-linear model used to fit the height–SD relationships per species fitted the data well, with a markedly more rapid height gain with increasing stem diameter in forest species (Supplementary Data Fig. S2). Likewise, models used to predict OBT based on SD fitted the data very well (0.57 ≤*r*^2^ ≤0.93; Supplementary Data Fig. S3), but three species had low goodness-of-fit metrics (0.11 ≤ *r*^2^ ≤ 0.25; Fig. S3A, D, Q) due to outliers suggesting OB loss. Models used to fit IB vs SD fitted the data very well for all species (Supplementary Data Fig. S4; 0.74 ≤ *r*^2^ ≤ 0.98).

**Fig. 7.**
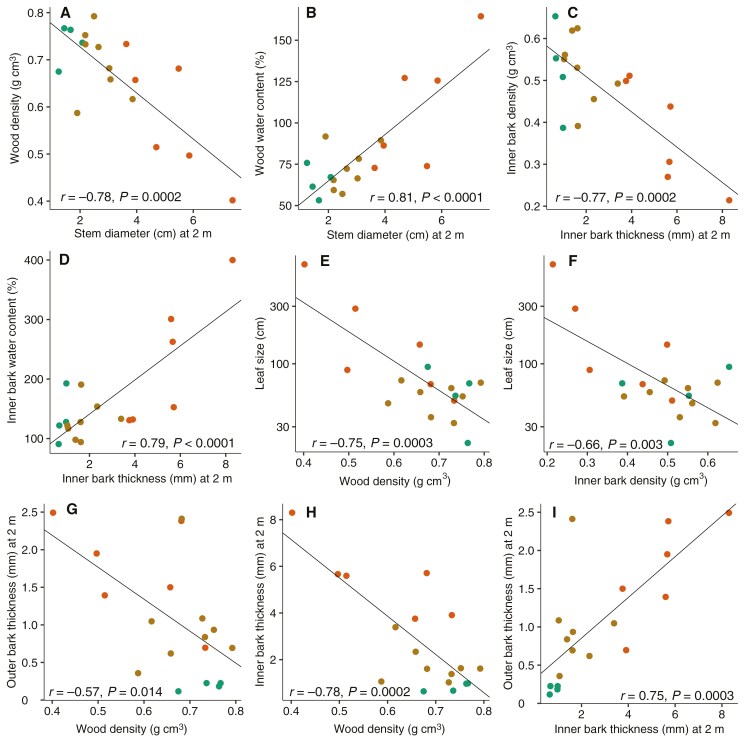
Bivariate relationships showing covariation between wood and bark density and water content, leaf length, and stem diameter, inner and outer bark thickness at 2 m in height. Closed-canopy species in green, clump-forming species in brown and savanna species in orange.

There was significant covariation between bark, stem and leaf traits (Fig. 7). Values of SD_2m_ were negatively correlated with sapwood density (*r* = −0.78, *P* < 0.001; Fig. 7A) and positively with wood water content (*r* = 0.81, *P* < 0.001; Fig. 7B). Likewise, IBT_2m_ was negatively correlated with IB density (*r* = −0.77, *P* < 0.001; Fig. 7C) and positively with IB water content (*r* = 0.79, *P* < 0.001; Fig. 7D). Closed-canopy species had the densest tissues, the lowest water contents, the thinnest stems and IB at 2 m height, with savanna species having the opposite trait values. Clump-forming species had intermediate IBT_2m_ and SD_2m_, while their sapwood and IB density and water content occupied a similar range to those of closed-canopy species. Species with larger leaves had lower sapwood (*r* = −0.75, *P* < 0.001; Fig. 7E) and IB density (*r* = −0.66, *P* < 0.005; Fig. 7F). Wood density was negatively correlated with OB_2m_ (*r* = −0.57, *P* < 0.05; Fig. 7G) and even more strongly with IBT_2m_ (*r* = −0.78, *P* < 0.001; Fig. 7H). There was a positive correlation between IBT_2m and_ OBT_2m_ (*r* = 0.75, *P* < 0.001; Fig. 7I).

**Fig. 8.**
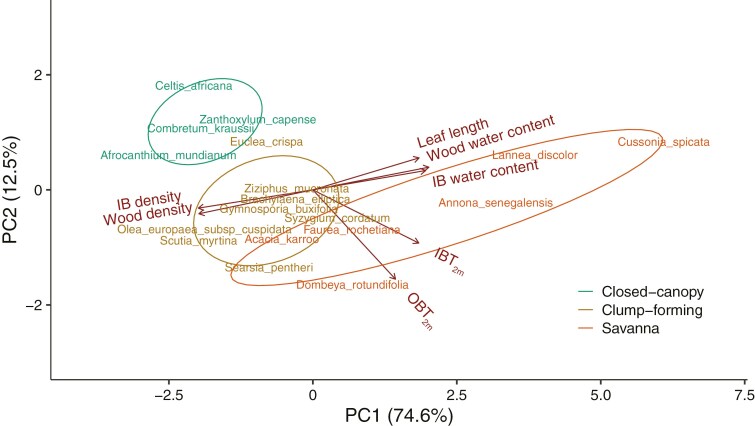
Principal component analysis for IB density and water content, wood density and water content, leaf length, inner bark (IBT_2m_) and outer bark thickness (OBT_2m_) for individuals 2 m in height.

The first three principal component axes explained 94 % of the variation in the seven traits (Supplementary Data Table S6, Fig. 8). The first principal component (PC1) explained 74.6 % of variation and had high loadings for water content of IB and wood, leaf length and IB_2m_, all covarying in the same direction, and for IB and wood density, which covaried negatively with the previous variables (Fig. 8). Species of the savanna were scattered along PC1, reflecting a wide range in density and leaf length within this group. In contrast, closed-canopy and clump-forming species were located in the quadrant of higher tissue density, lower water content and smaller leaf size (Fig. 8). The second principal component (PC2) explained 12.5 % of the variation. IBT_2m_ and OBT_2m_ had high loadings in this component, variables that covaried positively (Supplementary Data Table S6). Along PC2, closed-canopy species were located at the extreme of thin IB and OB, whereas savanna and clump-forming species overlapped in their range of variation for IBT_2m_ and OBT_2m_.

## DISCUSSION

Our results strongly suggest that high OB investment is crucial for bush clump initiation due to the protection it provides stems against fire, allowing saplings to attain the fire-resistance threshold (Hoffmann *et al.*, 2012). Although total bark is commonly regarded as the main trait protecting stems from fire (Pausas, 2015; Charles‐Dominique *et al.*, 2017), recent studies point to OB as the main region providing this protection (Rosell, 2016; Loram-Lourenço *et al.*, 2020; Scalon *et al.*, 2020, 2021). In our dataset, clump-forming species showed allocation to OB for a given SD that was similar to savanna species, which is congruent with OB providing protection against fire while establishing a clump around a savanna tree. Allocation to OB was statistically indistinguishable (equivalent slopes and intercepts) between clump-forming and savanna species in the semi-arid savanna (Supplementary Data Table S2). In contrast, in the mesic savanna we recovered differences in the allocation to OB for a given SD between the savanna and the clump-forming species, but the allocation was still much more similar between them than with the closed-canopy set (Supplementary Data Table S2). In both contrasting savannas examined here, OB was strongly associated with clump-forming species, in strong contrast to species establishing in fire-free forest or thicket, highlighting that fire must be a crucial selective factor in early stages of clump formation and at the edges of bush clumps.

Differences in the fire regime of the savanna being encroached affected OB allocation in the savanna set, but not in the clump-forming set. Mesic savannas, like the one at Sudwala, tend to experience greater fire intensity than drier ones, like the one at Endwell (Govender *et al.*, 2006), which explains the higher OB allocation in larger sized trees of the mesic savanna in comparison with the semi-arid one (Fig. 4C). In contrast with the savanna, OB thickness for a given SD was practically the same in the clump-forming species between sites (Fig. 4B). This similarity suggests that not only microclimate (Jamison-Daniels *et al.*, 2021) but also the effect of fire could be somewhat buffered for clump-forming individuals when they establish around savanna trees (Abreu *et al.*, 2021). Future studies examining the role of OB in clump formation will benefit from focusing on encroachment processes with a wider diversity of species and climatic conditions.

Our results indicated that the IB–SD allocation slope was mainly driven by metabolic needs, whereas its intercept was mainly explained by environmental conditions. Comprising mostly living tissues, IB includes the photosynthate-translocating cells in the secondary phloem, plus the storage parenchyma in the phloem, the rays, the cortex and the phelloderm (Evert, 2006; Angyalossy *et al.*, 2016). We expected a similar IBT–SD scaling independently of ecological category, indicating metabolic proportionality between IB and living wood (Rosell *et al.*, 2017; Rosell, 2019). Similar IBT–SD scaling was indeed observed across ecological groups at Sudwala (Fig. 5B). Nevertheless, at Endwell, clump-forming species had a low slope that was strongly influenced by *Olea europaea* subsp. *africana*, which had a relatively thin IB for its large SD. Removing this species from the analysis led to homogeneous slopes (Supplementary Data Table S4), in further support of our hypothesis. The relatively thin IB of this species could be explained by the probably large amount of non-metabolically active heartwood in the stem of this, the largest species in our dataset (up to 34 cm in SD). Inner bark is expected to scale with the living tissues in wood, i.e. sapwood, but not with heartwood (Rosell *et al.*, 2017). Given that we examined scaling with SD without differentiating between sapwood and heartwood, species with larger diameters will inevitably exhibit thinner IB for a given SD, but scaling of IB with the living tissues in the stem is likely the same (cf. Berry *et al.*, 2024).

While similar IBT–SD slopes indicated similar metabolic proportionality across stem sizes, different intercepts across groups pointed to different storage demands in association with environmental conditions. We had predicted that the savanna and clump-forming groups would have higher IBT–SD intercepts, indicating that storage in the mostly parenchymatous tissue of IB would be favoured in the seasonally dry and fire-prone environment of savannas (Rosell and Olson, 2014; Loram-Lourenço *et al.*, 2020). This prediction was based on the observation that IB storage capacity seems more driven by variation in IB quantity than quality (Rosell *et al.*, 2021). As predicted, intercepts were higher for savanna and clump-forming species, which are exposed to seasonal drought and fire (Abreu *et al.*, 2021). Water and non-structural carbohydrates in IB can participate in refilling of drought-induced embolism (Nardini *et al.*, 2011; Rosell *et al.*, 2021). Moreover, these carbohydrates, along with stored nutrients, would also be crucial for producing new stems and leaves after fire (Jones *et al.*, 2019; Heineman *et al.*, 2021; Rosell *et al.*, 2023), a situation savanna and clump-forming species would be faced with regularly. Even though intercepts across ecological groups differed within sites, differences did not emerge between the two encroachment processes. This lack of differences could be explained by the fact that although precipitation at Endwell is lower than at Sudwala (730 vs 930 mm), Sudwala experiences a markedly drier dry season than Endwell, and the dry season length at the two sites is similar (Supplementary Data Fig. S1). As a result, species at both sites seem to have similar storage needs, which is reflected in similar IBT–SD intercepts (Fig. 6).

In addition to having thicker OB and IB, clump-forming and savanna species were predicted to grow quickly, attaining fire and drought resistance while conditions are favourable (Cardoso *et al.*, 2016). Although we did not measure growth rates directly, we examined traits that are known to covary strongly with faster growth rate, including lower tissue density, higher water content and larger leaf size (Ackerly and Donoghue, 1998; Olson *et al.*, 2018). Within this multivariate trait space, closed-canopy species formed a distinct group, occupying the high density, low water content, small leaf, and thin IB and OB extreme of the spectrum that is associated with a more resource-conservative ecological strategy (Fig. 8). In turn, savanna species occupied a greater range of this multivariate space (Figs 7 and 8), with several species having a more resource-acquisitive ecological strategy, with ‘cheaper’ tissues of lower density in association with larger leaves (Olson *et al.*, 2018). They also had thicker stems relative to height in our data, an additional attribute that has been documented in Brazilian savanna–forest comparisons (Flake *et al.*, 2021) and linked with a decreased probability of topkill (Higgins *et al.*, 2012). We expected trait values of clump-forming species to be close to savanna species in trait space. However, like the generalist species documented by Flake *et al.* (2021), clump-forming species were largely intermediate between closed-canopy and savanna species, being actually closer to closed-canopy species regarding density, water content and leaf size (PC1 in Fig. 8, Supplementary Data Table S6), although closer to savannas in IB and OB thickness (PC2 in Fig. 8, Supplementary Data Table S6). This intermediate condition is plausibly associated with the ability to survive in the savanna habitat and later to survive in a closed-canopy vegetation.

Managing woody encroachment requires understanding of the mechanisms underlying bush clump formation. Our study emphasizes that trait-based approaches can offer key information for this understanding. In particular, we show that bark traits are crucial in allowing species to establish and survive in a savanna landscape to form bush clumps that represent the first step in a biome shift to closed-canopy vegetation. These traits include thick OB providing protection against fire (Graves *et al.*, 2014; Schafer *et al.*, 2015; Rosell, 2016; Scalon *et al.*, 2021), and thick IB providing the storage of water, non-structural carbohydrates and nutrients that fuel resprouting and provide drought resistance (Loram-Lourenço *et al.*, 2020; Rosell *et al.*, 2021, 2023; Scalon *et al.*, 2021; Vázquez-Segovia *et al.*, 2025), e.g. by aiding in restoration of hydraulic pathways after embolism. Moreover, our data suggest a trait syndrome associated with clump initiation, including relatively high-density wood and bark, low tissue water content, relatively small leaf sizes, and thick IB and OB. The ecological significance of these traits requires further investigation but is consistent with clump-initiators (and possibly forest margin species) having to deal with equally frequent and less intense fire than savanna species, and intermediate shade and drought levels (Cardoso *et al.*, 2016). Presumably, the ability of a species to initiate a clump is conferred by its traits. Therefore, identifying the traits enabling species to encroach into savannas and initiate closed-canopy vegetation is crucial for understanding how savanna vegetation is responding to global change.

## SUPPLEMENTARY DATA

Supplementary data are available at *Annals of Botany* online and consist of the following.

Table S1: models predicting OBT based on SD and ecological categories (closed-canopy, clump-forming, savanna). Table S2: models for intraspecific allometries of OBT and SD between the two examined sites for the different ecological categories (closed-canopy, clump-forming, savanna). Table S3: models predicting IBT based on SD and ecological categories (closed-canopy, clump-forming, savanna). Table S4: models for intraspecific allometries of IBT and SD across ecological categories (closed-canopy, clump-forming, savanna) excluding *Olea europaea* subsp. *africana* from the Endwell analyses and the analyses of the sites pooled. Table S5: models for intraspecific allometries of IBT and SD between the two examined sites for the different ecological categories (closed-canopy, clump-forming, savanna). Table S6: explained variance and loadings for the first three principal components (PCs) of the principal component analysis for IB density and water content, wood density and water content, leaf length, IBT_2m_ and OBT_2m._ Figure S1: monthly precipitation for Endwell and Sudwala showing that total precipitation is much higher in Sudwala, but that the dry season is characterized by markedly lower precipitation than Endwell. Figure S2: models for height vs stem diameter relationships per species. These models were used to calculate SD for individuals 2 m in height. Figure S3: models for OBT vs SD relationships per species. Figure S4: models for IBT vs SD relationships per species.

mcaf019_suppl_Supplementary_Figures_S1-S4_Tables_S1-S6

## Data Availability

Data will be available in TRY (www.try-db.org) upon publication.
