# Supplementary material for: Bark investment is key to forest expansion into African savannas by conferring resistance to fire and seasonal drought
Source: Ann Bot. 2025 May 2;135(6):1215–28. doi: 10.1093/aob/mcaf019 (PMC12259531; doi:10.1093/aob/mcaf019)
Supplement: mcaf019_suppl_Supplementary_Figures_S1-S4_Tables_S1-S6 [file mcaf019_suppl_supplementary_figures_s1-s4_tables_s1-s6.docx]

**SUPPLEMENTARY INFORMATION**

**Bark investment is key to forest expansion into African savannas by conferring resistance to fire and seasonal drought**

Julieta A. Rosell^1,2*^, Susanne Vetter^2^, Mark E. Olson^2,3^, Michelle Greve^4^

^1^Laboratorio Nacional de Ciencias de la Sostenibilidad, Instituto de Ecología, Universidad Nacional Autónoma de México, Tercer Circuito s/n de Ciudad Universitaria, Ciudad de México 04510, México

^2^Department of Botany, Rhodes University, Makhanda 6140, South Africa

^3^Instituto de Biología, Universidad Nacional Autónoma de México, Tercer Circuito s/n de Ciudad Universitaria, Ciudad de México 04510, México

^4^Department of Plant and Soil Sciences, University of Pretoria, Pretoria 0028, South Africa

**Supplementary Table S1**. Models predicting outer bark thickness (OBT) based on stem diameter (SD) and ecological categories (closed-canopy, clump-forming, savanna). Estimates are shown for coefficients with 95% confidence intervals in parenthesis. ^*^P<0.05, ^**^P<0.01, ^***^P<0.001. ^a-c^ homogeneous groups for intercepts, ^x-z^ homogeneous groups for slopes.

|  | Endwell | Sudwala | Sites pooled |
| --- | --- | --- | --- |
| Final model | log_10_OBT ~ log_10_SD * Ecological category | log_10_OBT ~ log_10_SD * Ecological category | log_10_OBT ~ log_10_SD * Ecological category |
| n | 206 | 253 | 459 |
| Marginal r^2^ (fixed only) | 0.741 | 0.779 | 0.766 |
| Conditional r^2^ (random and fixed) | 0.793 | 0.857 | 0.833 |
| Equality of slopes test (SD * Ecological category interaction) | P<0.001 | P<0.001 | P<0.001 |
| Intercept test | P=0.002 | P=0.083 | P<0.001 |
| Closed-canopy slope | 0.338 (0.112, 0.563)^**, x^ | 0.357 (0.076, 0.638)^***, x^ | 0.349 (0.158, 0.540)^***, x^ |
| Clump-forming slope | 0.901 (0.759, 1.043)^***, y^ | 0.883 (0.649, 1.116)^***, y^ | 0.895 (0.758, 1.031)^***, y^ |
| Savanna slope | 0.794 (0.464, 1.125)^***, y^ | 1.256 (1.075, 1.438)^***, z^ | 1.186 (1.029, 1.342)^***, z^ |
| Closed-canopy intercept | -1.096 (-1.513, -0.679)^***, a^ | -1.238 (-1.624, -0.853)^*, a^ | -1.169 (-1.439, -0.899)^***, a^ |
| Clump-forming intercept | -1.350 (-1.615, -1.086)^***, a^ | -1.300 (-1.622, -0.978)^***, a^ | -1.330 (-1.524, -1.136)^***, a^ |
| Savanna intercept | -1.397 (-2.009, -0.784) ^***, a^ | -1.897 (-2.152, -1.642)^***, b^ | -1.823 (-2.050, -1.597)^***, b^ |

**Supplementary Table S2**. Models for intraspecific allometries of outer bark thickness (OBT) and stem diameter (SD) between the two examined sites for the different ecological categories (closed-canopy, clump-forming, savanna). Estimates are shown for coefficients with 95% confidence intervals in parenthesis. ^***^P<0.001.

|  | Closed-canopy | Clump-forming | Savanna |
| --- | --- | --- | --- |
| Final model | log_10_OBT ~ log_10_SD + Site | log_10_OBT ~ log_10_SD + Site | log_10_OBT ~ log_10_SD * Site |
| n | 105 | 202 | 152 |
| Marginal r^2^ (fixed only) | 0.309 | 0.737 | 0.820 |
| Conditional r^2^ (random and fixed) | 0.399 | 0.863 | 0.843 |
| Equality of slopes test (SD * Site interaction) | P=0. 867 | P=0.864 | P=0.042 |
| Equality of intercepts | P=0.310 | P=0.817 | P=0.027 |
| Endwell intercept | -1.182 (-1.341, -1.022)^***^ | -1.344 (-1.542, -1.146)^***^ | -1.397 (-2.134, -0.659)^***^ |
| Endwell slope | 0.359 (0.214, 0.504)^***^ | 0.895 (0.782, 1.009)^***^ | 0.794 (0.389, 1.199)^***^ |
| Sudwala intercept | -1.182 (-1.341, -1.022)^***^ | -1.310 (-1.549, -1.069)^***^ | -1.893 (-2.206, -1.581)^***^ |
| Sudwala slope | Same as Endwell | Same as Endwell | 1.249 (1.082, 1.416)^***^ |

**Supplementary Table S3**. Models predicting inner bark thickness (IBT) based on stem diameter (SD) and ecological categories (closed-canopy, clump-forming, savanna). Estimates are shown for coefficients with 95% confidence intervals in parenthesis. ^*^P<0.05, ^**^P<0.01, ^***^P<0.001. ^a-c^ homogeneous groups for intercepts, ^x,y^ homogeneous groups for slopes

|  | Endwell | Sudwala | Sites pooled |
| --- | --- | --- | --- |
| Final model | log_10_IBT ~ log_10_SD * Ecological category | log_10_IBT ~ log_10_SD + Ecological category | log_10_IBT ~ log_10_SD + Ecological category |
| n | 206 | 253 | 459 |
| Marginal r^2^ (fixed only) | 0.828 | 0.890 | 0.854 |
| Conditional r^2^ (random and fixed) | 0.916 | 0.940 | 0.931 |
| Equality of slopes test (SD * Ecological category interaction) | P=0.030 (P=0.072 without *Olea europaea*) | P=0.086 | P=0.094 |
| Equality of intercepts | P=0.082 | P<0.001 | P<0.001 |
| Closed-canopy slope | 0.621 (0.509, 0.733)^***, x,y^ | 0.661 (0.585, 0.737)^***^ | 0.611 (0.506, 0.716)^***^ |
| Clump-forming slope | 0.505 (0.434, 0.575)^***, x^ | Same as closed canopy | Same as closed canopy |
| Savanna slope | 0.715 (0.551, 0.877)^***, y^ | Same as closed canopy | Same as closed canopy |
| Closed-canopy intercept | -0.750 (-0.890, -0.610)^***, a^ | -0.946 (-1.083, -0.808)^***, a^ | -0.811 (-0.974, -0.648)^***, a^ |
| Clump-forming intercept | -0.516 (-0.605, -0.428)^***, b^ | -0.712 (-0.833, -0.590)^***, b^ | -0.639 (-0.792, -0.486)^***, b^ |
| Savanna intercept | -0.524 (-0.733, -0.315) ^***, a,b^ | -0.422 (-0.530, -0.315)^***, c^ | -0.377 (-0.535, -0.219)^***, c^ |

**Supplementary Table S4**. Models for intraspecific allometries of inner bark thickness (IBT) and stem diameter (SD) across ecological categories (closed-canopy, clump-forming, savanna) excluding *Olea europaea* subsp. *africana* from the Endwell analyses and the analyses of the sites pooled. The model for Sudwala is the same as that presented in Supplementary Table S2. Estimates are shown for coefficients with 95% confidence intervals in parenthesis. ^*^P<0.05, ^**^P<0.01, ^***^P<0.001. ^a-c^ homogeneous groups for intercepts, ^x,y^ homogeneous groups for slopes.

|  | Endwell | Sudwala | Sites pooled |
| --- | --- | --- | --- |
| Final model | log_10_IBT ~ log_10_SD + Ecological category | log_10_IBT ~ log_10_SD + Ecological category | log_10_IBT ~ log_10_SD + Ecological category |
| n | 180 | 253 | 433 |
| Marginal r^2^ (fixed only) | 0.784 | 0.890 | 0.856 |
| Conditional r^2^ (random and fixed) | 0.914 | 0.940 | 0.931 |
| Equality of slopes test (SD * Ecological category interaction) | P=0.072 | P=0.086 | P=0.099 |
| Equality of intercepts | P=0.103 | P<0.001 | P<0.001 |
| Closed-canopy slope | 0.577 (0.500, 0.653)^***^ | 0.661 (0.585, 0.737)^***^ | 0.620 (0.529, 0.711)^***^ |
| Clump-forming slope | Same as closed-canopy | Same as closed-canopy | Same as closed-canopy |
| Savanna slope | Same as closed-canopy | Same as closed-canopy | Same as closed-canopy |
| Closed-canopy intercept | -0.731 (-0.850, -0.612)^***, a^ | -0.946 (-1.083, -0.808)^***, a^ | -0.817 (-0.961, -0.673)^***, a^ |
| Clump-forming intercept | -0.568 (-0.655, -0.480)^***, a^ | -0.712 (-0.833, -0.590)^***, b^ | -0.653 (-0.787, -0.519)^***, b^ |
| Savanna intercept | -0.459 (-0.632, -0.285) ^***, a^ | -0.422 (-0.530, -0.315)^***, c^ | -0.390 (-0.529, -0.252)^***, c^ |

**Supplementary Table S5**. Models for intraspecific allometries of inner bark thickness (IBT) and stem diameter (SD) between the two examined sites for the different ecological categories (closed-canopy, clump-forming, savanna). Estimates are shown for coefficients with 95% confidence intervals in parenthesis. ^***^P<0.001.

|  | Closed-canopy | Clump-forming | Savanna |
| --- | --- | --- | --- |
| Final model | log_10_IBT ~ log_10_SD | log_10_IBT ~ log_10_SD | log_10_IBT ~ log_10_SD |
| n | 105 | 202 | 152 |
| Marginal r^2^ (fixed only) | 0.851 | 0.781 | 0.924 |
| Conditional r^2^ (random and fixed) | 0.886 | 0.914 | 0.936 |
| Equality of slopes test (SD * Site interaction) | P=0.111 | P=0.051 | P=0.919 |
| Equality of intercepts | P=0.088 | P=0.443 | P=0.453 |
| Endwell intercept | -0.779 (-0.844, -0.715)^***^ | -0.595 (-0.721, -0.470)^***^ | -0.481 (-0.555, -0.407)^***^ |
| Endwell slope | 0.573 (0.488, 0.658)^***^ | 0.570 (0.468, 0.672)^***^ | 0.709 (0.666, 0.752)^***^ |
| Sudwala intercept | Same as Endwell | Same as Endwell | Same as Endwell |
| Sudwala slope | Same as Endwell | Same as Endwell | Same as Endwell |

**Supplementary Table S6**. Explained variance and loadings for the first three principal components (PC) of the principal component analysis for IB density and water content, wood density and water content, leaf length, IB (IBT_2m_) and OB thickness (OBT_2m_) for individuals 2m in height.

|  | PC1 | PC2 | PC3 |
| --- | --- | --- | --- |
| Explained variance (%) | 74.6 | 12.5 | 6.9 |
| Cumulative explained variance (%) | 74.6 | 87.0 | 93.9 |
| IB density | -0.401 | -0.157 | 0.537 |
| IB water content | 0.395 | 0.166 | -0.573 |
| Wood density | -0.399 | -0.205 | -0.355 |
| Wood water content | 0.406 | 0.194 | 0.391 |
| leaf length | 0.372 | 0.274 | 0.314 |
| IBT_2m_ | 0.371 | -0.456 | 0.016 |
| OBT_2m_ | 0.289 | -0.765 | 0.063 |

**Supplementary Figure S1**. Monthly precipitation for Endwell and Sudwala showing that total precipitation is much higher in Sudwala, but that the dry season is characterized by markedly lower precipitation than Endwell.

**Supplementary Figure S2**. Models for height vs. stem diameter relationships per species. These models were used to calculate stem diameter for individuals with 2m in height. Closed-canopy species in green, clump-forming species in brown, savanna species in orange.

**Supplementary Figure S2**. cont.

**Supplementary Figure S2**. cont.

**Supplementary Figure S3**. Models for outer bark thickness (OBT) vs. stem diameter (SD) relationships per species. These models were used to calculate OBT for individuals with 2m in height. Closed-canopy species in green, clump-forming species in brown, savanna species in orange.

**Supplementary Figure S3** cont.

**Supplementary Figure S3** cont.

**Supplementary Figure S4**. Models for inner bark thickness (IBT) vs. stem diameter (SD) relationships per species. These models were used to calculate IBT for individuals with 2m in height. Closed-canopy species in green, clump-forming species in brown, savanna species in orange.

**Supplementary Figure S4** cont.

**Supplementary Figure S4**. Cont.
